# Assembly Mechanism for Aggregation of Amyloid Fibrils

**DOI:** 10.3390/ijms19072141

**Published:** 2018-07-23

**Authors:** Lingyun Zhang

**Affiliations:** Beijing National Laboratory for Condensed Matter Physics, Institute of Physics, Chinese Academy of Sciences, Beijing 100190, China; lyzhang@iphy.ac.cn

**Keywords:** amyloid fibrils, assembly mechanism, partition function, free energy

## Abstract

The assembly mechanism for aggregation of amyloid fibril is important and fundamental for any quantitative and physical descriptions because it needs to have a deep understanding of both molecular and statistical physics. A theoretical model with three states including coil, helix and sheet is presented to describe the amyloid formation. The corresponding general mathematical expression of N molecule systems are derived, including the partition function and thermodynamic quantities. We study the equilibrium properties of the system in the solution and find that three molecules have the extreme value of free energy. The denaturant effect on molecular assemble is also discussed. Furthermore, we apply the kinetic theories to take account of the nucleation and growth of the amyloid in the solution. It has been shown that our theoretical results can be compared with experimental results.

## 1. Introduction

The aggregation of amyloid fibrils in biological processes is associated with neurodegenerative diseases such as Alzheimer’s, Parkinson’s and Huntington’s or prion diseases [1,2,3]. Despite the specificity of the proteins related with each individual neurodegenerative disease, these kinds of diseases are a nucleation process [4,5,6,7,8,9]. Under experimental conditions in vitro, the aggregation pathway can be obtained. However, it is not clear how to extrapolate these results to identify the dominant pathway and timescales under physiological conditions.

The current computation technique is unable to access even the accelerated timescales of the in vitro systems. Some computational techniques could be used to predict the assembly of amyloid in solution and their secondary structure changes [10,11]. However, these computational simulations are only feasible for millisecond time scale. The most simplistic physical description of proteins is analogy with colloidal particles. The random-coil like proteins exist in an unfolded state and the helix is very similar to folded state. These simple models have been used to explore the nucleation processes of amyloid fibrils. There are two classes of nucleation theories, one is the mass action theories, the other is nucleation models [12,13,14,15,16,17,18,19,20,21]. However, these methods missed the internal dynamics of the protein molecules. In this paper, a microscopic model of the assembly process is developed to explore the mechanism of amyloid fibrils and explain the transitions between the various assembly pathways as well as how side chain interactions determine the sheet structure in the aggregate phase. Based on the microscopic model, molecular equilibrium states and kinetic equations have been constructed to probe the physical properties of amyloid formation. The paper is organized as follow. Firstly, a single peptide molecule in the solution can be transited from a coil state to a helix state. Then two molecules will be formed by concentration in the solution that supplies a driving force. The combining force is resulted from the hydrogen bond. Based on the structure of two molecules, three molecules can be constructed with the aid of solution concentration. Furthermore, N molecule systems are able to be constructed, which is called β-sheet. The corresponding general mathematical description of N molecule systems are derived, including the partition function and thermodynamic quantities. Then we study the equilibrium properties of the system in the solution. The phase diagrams of assembly structures are depicted. Furthermore, we employ the kinetic theories to study the amyloid formation. Free energy landscape and side chain effect can be illustrated. The theoretical predictions are in agreement with the experimental results.

## 2. Results

### 2.1. Single Molecule

The conformations in single molecule have sequences of helix and coil units [22]. The coil state is an ensemble of disordered conformations which often exists for higher temperature, otherwise, the helix state exists for lower temperature [23]. Therefore, the temperature determines the transition between coil and helix. The experimental temperature ranges from 10 to 60 °C [24]. This structural transition is a cooperative behavior [25,26].

In order to provide a valuable intuition into the nucleation process, we use a similar lattice model and suppose a single molecular chain has *N* units, each unit along the chain can be in either of the two states, *H* (helix) or *C* (coil). According to the ZB model [27,28], only the nearest neighbor interaction is considered, which is a modified one-dimensional Ising model. Therefore, there are four statistical weights, their matrix elements are $m_{11}=m\left(C|C\right),m_{12}=m\left(H|C\right),m_{21}=m\left(C|H\right),m_{22}=m\left(H|H\right)$. A propagation parameter *s* can be defined by the free energy change,

(1) $$s=\exp[-\beta \left(F_{helix}-F_{coil}\right)]$$

where *s* is slightly larger than 1, so the matrix element m(H|H)=s. The other parameter σ is as a nucleation parameter. In general, σ<<1 and takes from $10^{-3}$ to $10^{-4}$, so σs is for helix next to coil, i.e., m(H|C)=σs. On the other hand, one uses the statistical weight 1 for any coil, and m(C|C)=m(C|H)=1. Then a matrix form of statistical weight can be defined as

(2) $$M=\left(\begin{array}{cc} 1 & \sigma s\\ 1 & s \end{array}\right)$$

Furthermore, the partition function can be obtained by summing over all the possible sequences in the one-dimensional chain with *N* monomers,

(3) $$Z_{N}=\left(\begin{array}{cc} 1 & 0 \end{array}\right)\cdot M^{N}\cdot \left(\begin{array}{c} 1\\ 1 \end{array}\right)$$

Let *T* be a transformation matrix to get a diagonalize matrix Λ,

(4) $$T^{-1}MT=\Lambda =\left(\begin{array}{cc} \lambda_{1} & 0\\ 0 & \lambda_{2} \end{array}\right)$$

where $\lambda_{1},\lambda_{2}$ are the eigenvalues of *M*.

(5) $$\lambda_{1,2}=\frac{1}{2}\left[\left(1+s\right)\pm \sqrt{{\left(1-s\right)}^{2}+4s\sigma }\right]$$

By employing $M^{N}={\left(TT^{-1}MTT^{-1}\right)}^{N}=T\Lambda^{N}T^{-1}$, the partition function can be rewritten as

(6) $$Z_{N}=\frac{1}{\lambda_{1}-\lambda_{2}}\left[\lambda_{1}^{N+1}\left(1-\lambda_{2}\right)-\lambda_{2}^{N+1}\left(1-\lambda_{1}\right)\right]$$

Because of $\lambda_{1}>\lambda_{2}$, we have $\lambda_{1}^{N+1}>>\lambda_{2}^{N+1}$ for large *N*, then

(7) $$Z_{N}\approx \lambda_{1}^{N}$$

Based on $\ln Z_{N}=N\ln\lambda_{1}$, the average number of helix states can be computed by

(8) $$<h>=\frac{\partial \ln Z_{N}}{\partial \ln s}=N\frac{\partial \ln\lambda_{1}}{\partial \ln s}$$

and the fraction of helix states $\theta_{h}$ is defined as

(9) $$\theta_{h}=\frac{<h>}{N}=\frac{s}{2\lambda_{1}}\left\{1+\frac{\left[(s-1)+2\sigma \right]}{\sqrt{{\left(1-s\right)}^{2}+4\sigma s}}\right\}$$

The free energy for the single molecule can approximately be expressed as

(10) $$F^{\left(1\right)}=-k_{B}T\ln Z_{N}=-Nk_{B}T\ln\lambda_{1}$$

**Figure 1:**
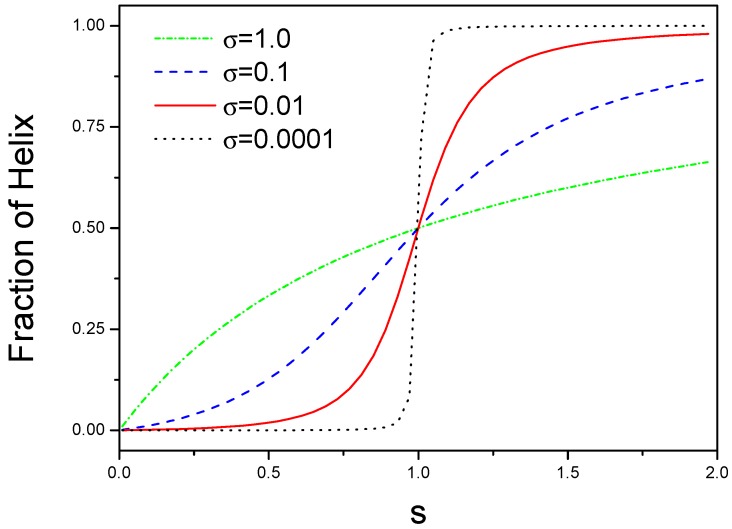
Phase diagrams of structural transition from coil to helix for different interactions. The number of helical state in the block is plotted against *s*, where *s* is an equilibrium constant for a coil state converting into a helical state.

We plot $\theta_{h}$ as a function of *s* for different σ as shown in Figure 1. There are two limitation cases for σ-value. For small σ, a sudden transition can occur for a narrow range of *s*, this is a helix-coil structural transition. For large σ, there is a non-cooperative behavior because $\theta_{h}$ is equal to s/(1+s) for σ=1.

### 2.2. Two Molecules

**Figure 2:**
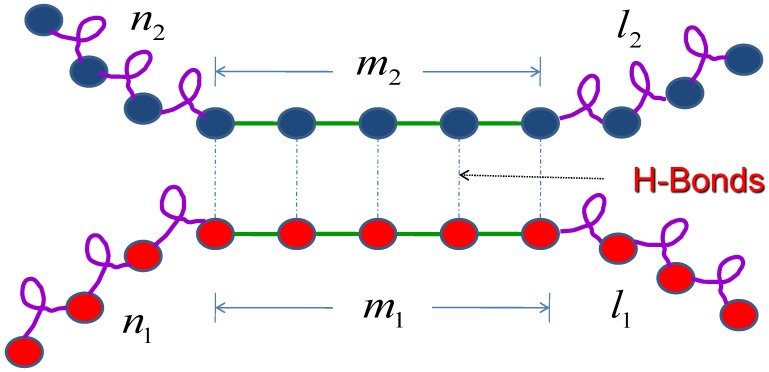
Schematic illustration of the monomer attachment. Two molecules through intermolecular hydrogen bond form a simplistic helix-coil-sheet model. $m_{1}$ and $m_{2}$ are the total number of hydrogen bonds for molecule 1 and molecule 2 respectively.

There are two cases for two molecules, one is no interaction between two molecules, and the other case is the interaction between two molecules. The partition function can be written as

(11) $$Z^{\left(2\right)}=\sum_{\left\{m_{1},m_{2}\right\}}\Omega^{\left(2\right)}\left(m_{1},m_{2}\right)Z^{\left(2\right)}\left(m_{1},m_{2}\right)$$

where $\Omega^{\left(2\right)}\left(m_{1},m_{2}\right)$ is the degeneracy factor,

(12) $$\Omega^{\left(2\right)}\left(m_{1},m_{2}\right)=\left(N-m_{1}\right)\left(N-m_{2}\right)$$

which represents that $N-m_{1}$ hydrogen bonds in the first molecular chain and $N-m_{2}$ hydrogen bonds in the second molecular chain. The other factor in the above $Z^{\left(2\right)}\left(m_{1},m_{2}\right)$ is

(13) $$Z^{\left(2\right)}\left(m_{1},m_{2}\right)=Z_{n_{1}}Z_{l_{1}}g_{2}^{m_{2}}Z_{n_{2}}Z_{l_{2}}$$

where $g_{2}$ is the interaction parameter between the first molecule and the second molecule, which results from the hydrogen bond, $m_{1}$ and $m_{2}$ are the total number of hydrogen bond of molecule 1 and molecules 2 respectively. $Z_{n_{1}}$, $Z_{l_{1}}$, $Z_{n_{2}}$ and $Z_{l_{2}}$ are the partition functions for each segment as shown in Figure 2.

By defining the parameters $\theta_{1}$ and $\theta_{2}$ to demonstrate the ratio of non-interaction,

(14) $$\begin{array}{c} \theta_{1}=\frac{Z_{n_{1}}Z_{l_{1}}}{Z_{N}}\\ \theta_{2}=\frac{Z_{n_{2}}Z_{l_{2}}}{Z_{N}} \end{array}$$

then the partition function of the two molecules can be rewritten as

(15) $$Z^{\left(2\right)}=\sum_{\left\{m_{1},m_{2}\right\}}\left(N-m_{1}\right)\left(N-m_{2}\right)Z_{N}^{2}\theta_{1}g_{2}^{m_{2}}\theta_{2}$$

Actually, we are able to discuss how chain length influences the partition function. According to the derivation for the single molecule, the partition function can be simplified as

(16) $$Z_{n_{1}}\approx \lambda_{1}^{n_{1}}\;\;\;\;\;\;\;Z_{l_{1}}\approx \lambda_{1}^{l_{1}}$$

then $\theta_{1}$ is

(17) $$\theta_{1}=\lambda_{1}^{n_{1}+l_{1}-N}=\lambda_{1}^{-m_{1}}$$

Due to the same reason, $\theta_{2}$ is

(18) $$\theta_{2}=\lambda_{1}^{-m_{2}}$$

thus we have

(19) $$Z^{\left(2\right)}\left(m_{1},m_{2}\right)=Z_{N}^{2}\lambda_{1}^{-m_{1}-m_{2}}g_{2}^{m_{2}}$$

In terms of $m_{1}=m_{2}=m$, we obtain

(20) $$Z^{\left(2\right)}\left(m\right)=Z_{N}^{2}{\left(\frac{g_{2}}{\lambda_{1}^{2}}\right)}^{m}$$

Therefore, the partition function of two molecules can be rewritten as

(21) $$Z^{\left(2\right)}=Z_{N}^{2}\sum_{m=1}^{M}{\left(N-m\right)}^{2}{\left(\frac{g_{2}}{\lambda_{1}^{2}}\right)}^{m}$$

where *M* is the total number of the hydrogen bond, its maximum value is $M_{max}=N$.

A function can be defined as

(22) $$G_{2}\left(m,x_{2}\right)=x_{2}^{m}$$

where $x_{2}=g_{2}/\lambda_{1}^{2}$. The other function can be defined as

(23) $$f^{\left(2\right)}\left(M,x_{2}\right)=\frac{Z^{\left(2\right)}}{Z_{N}^{2}}=\sum_{m=1}^{M}{\left(N-m\right)}^{2}G_{2}\left(m,x_{2}\right)$$

The free energy of two molecules is obtained by

(24) $$F^{\left(2\right)}=-k_{B}T\ln Z^{\left(2\right)}=2F^{\left(1\right)}-k_{B}Tlnf^{\left(2\right)}\left(M,x_{2}\right)$$

### 2.3. Three Molecules

Now we add the third molecule to the dimer to form a trimer. The partition function for this system can be represented as

(25) $$Z^{\left(3\right)}=\sum_{m_{3}\leq m_{2}}\sum_{m_{2}}\Omega^{\left(3\right)}\left(m_{2},m_{3}\right)Z^{\left(3\right)}\left(m_{2},m_{3}\right)$$

where the degeneracy factor for three molecules

(26) $$\Omega^{\left(3\right)}\left(m_{2},m_{3}\right)=2\left(m_{2}-m_{3}\right){\left(N-m_{2}\right)}^{2}$$

and

(27) $$\begin{array}{c} Z^{\left(3\right)}\left(m_{2},m_{3}\right)=Z^{\left(2\right)}\left(m_{2}\right)g_{3}^{m_{3}}Z_{n_{3}}Z_{l_{3}}\\ =Z_{N}^{3}\left[\frac{Z^{\left(2\right)}\left(m_{2}\right)}{Z_{N}^{2}}g_{3}^{m_{3}}\frac{Z_{n_{3}}Z_{l_{3}}}{Z_{N}}\right] \end{array}$$

where $Z_{n_{3}}$ and $Z_{l_{3}}$ are the partition function for the segments of $n_{3}$ and $l_{3}$ respectively.

Furthermore, we can define $\theta_{3}=Z_{n_{3}}Z_{l_{3}}/Z_{N}$. In a similar manner, $\theta_{3}\approx \lambda_{1}^{-m_{3}}$, we have

(28) $$Z^{\left(3\right)}\left(m,m_{3}\right)=Z_{N}^{3}G_{2}\left(m,x_{2}\right){\left(\frac{g_{3}}{\lambda_{1}}\right)}^{m_{3}}$$

We define a new function

(29) $$G_{3}\left(m_{3},m,x_{3},x_{2}\right)=G_{2}\left(m,x_{2}\right){x_{3}}^{m_{3}}$$

where $x_{3}=g_{3}/\lambda_{1}$. Then the partition function of three molecules can be rewritten as

(30) $$Z^{\left(3\right)}=2Z_{N}^{3}\sum_{m_{3}\leq m}\sum_{m}\left(m-m_{3}\right){\left(N-m\right)}^{2}G_{3}\left(m_{3},m,x_{3},x_{2}\right)$$

The free energy of three molecules can be obtained by

(31) $$F^{\left(3\right)}=-k_{B}T\ln Z^{\left(3\right)}=3F^{\left(1\right)}-k_{B}T\ln f^{\left(3\right)}\left(M_{3},M,x_{3},x_{2}\right)$$

### 2.4. General Expression of *n* Molecules

Now we extend the expressions of *n* molecules with β-strand, the first expression is the partition function of *n* molecules,

(32) $$\begin{array}{c} Z^{\left(n\right)}=\sum_{m_{n}\leq m_{n-1}}\sum_{m_{n-1}\leq m_{n-2}}\cdots \sum_{m_{3}\leq m_{2}}\sum_{m_{2}}\Omega^{\left(n\right)}\left(m_{2},m_{3},\cdots ,m_{n-1},m_{n}\right)\\ \times Z^{\left(n\right)}\left(m_{2},m_{3},\cdots ,m_{n-1},m_{n}\right) \end{array}$$

where the degeneracy factor of *n* molecules is

(33) $$\Omega^{\left(n\right)}\left(m_{2},m_{3},\cdots ,m_{n-1},m_{n}\right)=2\prod_{i=3}^{n}\left(m_{i-1}-m_{i}\right){\left(N-m_{2}\right)}^{2}$$

and

(34) $$Z^{\left(n\right)}\left(m_{2},m_{3},\cdots ,m_{n-1},m_{n}\right)=Z^{\left(n-1\right)}\left(m_{2},m_{3},\cdots ,m_{n-2},m_{n-1}\right)g_{n}^{m_{n}}Z_{nn}Z_{nl}$$

then $G_{n}$ function can be defined as

(35) $$G_{n}\left(m_{2},m_{3},\cdots ,m_{n-1},m_{n},x_{2},\cdots ,x_{n}\right)=G_{n-1}\left(m_{2},m_{3},\cdots ,m_{n-2},m_{n-1},x_{2},\cdots ,x_{n-1}\right)x_{n}^{m_{n}}$$

Let us define the function of $f^{\left(n\right)}$ as

(36) $$\begin{array}{c} f^{\left(n\right)}\left(M_{n},M_{n-1},\cdots ,M,x_{n},x_{n-1},\cdots ,x_{2}\right)=2\sum_{m_{n}=1}^{M_{n}}\cdots \sum_{m_{2}=1}^{M}\prod_{i=3}^{n}\left(m_{i-1}-m_{i}\right){\left(N-m_{2}\right)}^{2}\\ \times G_{n}\left(m_{2},m_{3},\cdots ,m_{n-1},m_{n},x_{2},\cdots ,x_{n}\right) \end{array}$$

where $x_{n}=g_{n}/\lambda_{1}$, and used $\theta_{n}\approx \lambda_{1}^{-m_{n}}$, so the partition function of *n* molecules can be rewritten as

(37) $$Z^{\left(n\right)}=Z_{N}^{n}f^{\left(n\right)}\left(M_{n},M_{n-1},\cdots ,M,x_{n},x_{n-1},\cdots ,x_{2}\right)$$

The expression of free energy is

(38) $$\begin{array}{c} F^{\left(n\right)}=-k_{B}T\ln Z^{\left(n\right)}\\ =nF^{\left(1\right)}-k_{B}T\ln f^{\left(n\right)}\left(M_{n},M_{n-1},\cdots ,M,x_{n},x_{n-1},\cdots ,x_{2}\right) \end{array}$$

As we mentioned, the reference state is the coil state which has a statistical weight of 1 (free energy =0). The helix state is favorable s>1 and the nucleation parameter is unfavorable 0<σ<1. A single β-sheet is probably not stable in solution [29,30], so g≈1 or slightly bigger than 1. However, a β-sheet bilayer is more stable than a helix, thus we need to introduce a new parameter, *z*, to describe side chain interactions, then gz>s. The free energy of β-sheet includes the conformation entropy (which supplies the repulsive force) and the interaction of hydrogen bond and the side-chain interactions (that is attractive force). Therefore, $x_{i}$ can be replaced by $x_{i,j}=x_{i}z_{i}^{j-1}$, the corresponding general expression of physics quantities for fibril structure with jβ-sheet can be written as

(39) $$\begin{array}{c} Z_{j}^{\left(n\right)}=Z_{N}^{n}f_{j}^{\left(n\right)}\left(z_{n},M_{n},M_{n-1},\cdots ,M,x_{n,j},x_{n-1,j},\cdots ,x_{2,j}\right)\\ F_{j}^{\left(n\right)}=nF^{\left(1\right)}-k_{B}T\ln f_{j}^{\left(n\right)}\left(M_{n},M_{n-1},\cdots ,M,x_{n,j},x_{n-1,j},\cdots ,x_{2,j}\right) \end{array}$$

where j=1,2 and

(40) $$\begin{array}{c} f_{j}^{\left(n\right)}\left(M_{n},M_{n-1},\cdots ,M,x_{n,j},x_{n-1,j},\cdots ,x_{2,j}\right)=2\sum_{m_{n}=1}^{M_{n}}\cdots \sum_{m_{2}=1}^{M}\prod_{i=3}^{n}\left(m_{i-1}-m_{i}\right){\left(N-m_{2}\right)}^{2}\\ \times G_{n}^{j}\left(m_{2},m_{3},\cdots ,m_{n-1},m_{n},x_{2,j},\cdots ,x_{n,j}\right) \end{array}$$

## 3. Discussion

### 3.1. Molecular Interaction with Denaturant

The influence of denaturant on amyloid fibril is investigated by the interaction parameter $x_{2}$. When the denaturant urea is added in the system, the free energy is dependent on the denaturant concentration, and can be expanded as the first order approximation,

(41) $$\Delta F_{i}=\Delta F_{i}\left(C_{urea}=0\right)+\delta_{i}C_{urea}$$

where $\delta_{i}>0$.

The parameters *s* and $g_{2}$ are expressed as

(42) $$\begin{array}{c} s=\exp\left[-\Delta F_{s}\left(0\right)-\delta_{s}C_{urea}\right]=s\left(0\right)\exp\left(-\delta_{s}C_{urea}\right]\\ g_{2}=\exp\left[-\Delta F_{g}\left(0\right)-\delta_{g}C_{urea}\right]=g_{2}\left(0\right)\exp\left[-\delta_{g}C_{urea}\right] \end{array}$$

where $s\left(0\right)=\exp[-\Delta F_{s}\left(0\right)]$, $g_{2}\left(0\right)=\exp\left[-\Delta F_{g}\left(0\right)\right]$. s(0)>1 is for the helix state (folded state), and s(0)<1 is for the coil state (unfolded state).

We make an approximation $\delta_{s}\approx \delta_{g}=\delta$, the expression of $x_{2}$ is

(43) $$x_{2}=\frac{g_{2}}{\lambda_{1}^{2}}=\frac{4g_{2}\left(0\right)\exp\left(\delta C_{urea}\right)/s^{2}\left(0\right)}{{\left\{\left[\frac{\exp(\delta C_{urea})}{s(0)}+1\right]+\sqrt{{\left[\frac{\exp(\delta C_{urea})}{s(0)}-1\right]}^{2}+4\frac{\sigma }{s(0)}\exp\left(\delta C_{urea}\right)}\right\}}^{2}}$$

When σ/s(0)<<1,

(44) $$x_{2}=\frac{4g_{2}\left(0\right)\exp\left(\delta C_{urea}\right)/s^{2}\left(0\right)}{\{\left[\frac{\exp(\delta C_{urea})}{s(0)}+1\right]+|\frac{\exp(\delta C_{urea})}{s(0)}-1{\left|\right\}}^{2}}$$

If exp$\left(\delta C_{urea}\right)/s\left(0\right)<1$, it is that the strong folded state

(45) $$x_{2}\left(sf\right)=\frac{g_{2}\left(0\right)}{s^{2}\left(0\right)}\exp\left(\delta C_{urea}\right)$$

If exp$\left(\delta C_{urea}\right)/s\left(0\right)>1$, it is that the strong unfolded state

(46) $$x_{2}\left(suf\right)=g_{2}\left(0\right)\exp\left(-\delta C_{urea}\right)$$

Based on Equations (45) and (46), we can see how the denaturant affects the aggregation process. For strong folded state, the denaturant can strengthen the hydrophobic and hydrogen binding interactions. Otherwise, adding denaturant can weaken the interactions for strong unfolded state.

### 3.2. Free Energy

**Figure 3:**
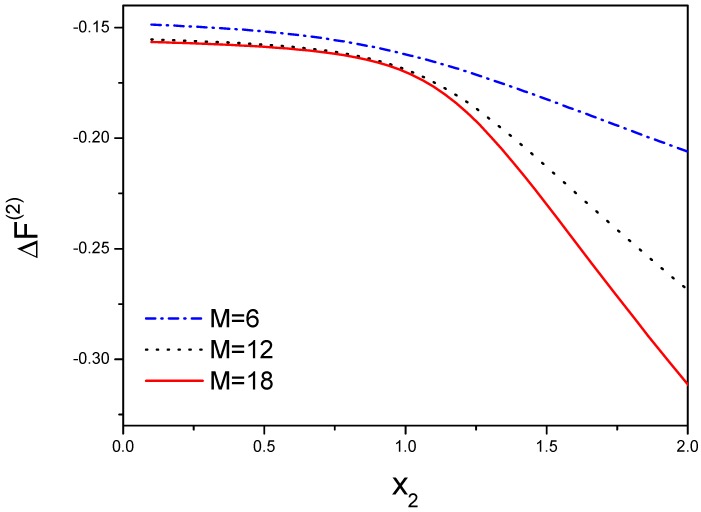
The free energy is dependent on the interaction between the first molecule and the second molecule. $x_{2}=g_{2}/\lambda_{1}^{2}$, where the free energy for each bond is $-k_{B}Tlng_{2}$ that illustrates the loss of conformational entropy from both chains, and $\lambda_{1}$ indicates the contribution form the peptide tails not participating in the hydrogen bonds. $\Delta F^{\left(2\right)}=F^{\left(2\right)}-2F^{\left(1\right)}$ accounts for the free energy difference from both chains. The free energy will be decreased when the number of the hydrogen bond increases from 6 to 18.

As shown in Figure 3, $\Delta F^{\left(2\right)}$ is a function of $x_{2}$ that represents the effect of $g_{2}$, σ and *s*. Here we choose N=20, M=18, *s* is from 0 to 2, θ is from 0.001 to 1 and $g_{2}$ is from 0 to 2. When $g_{2}$ is zero, there is not any interaction between two molecular chains. Thus $g_{2}$ is chosen as a smaller quantity, the interaction is a repulsive force, otherwise, the attractive force for larger $g_{2}$, so the range of $x_{2}$ is from $10^{-5}$ to 2.

**Figure 4:**
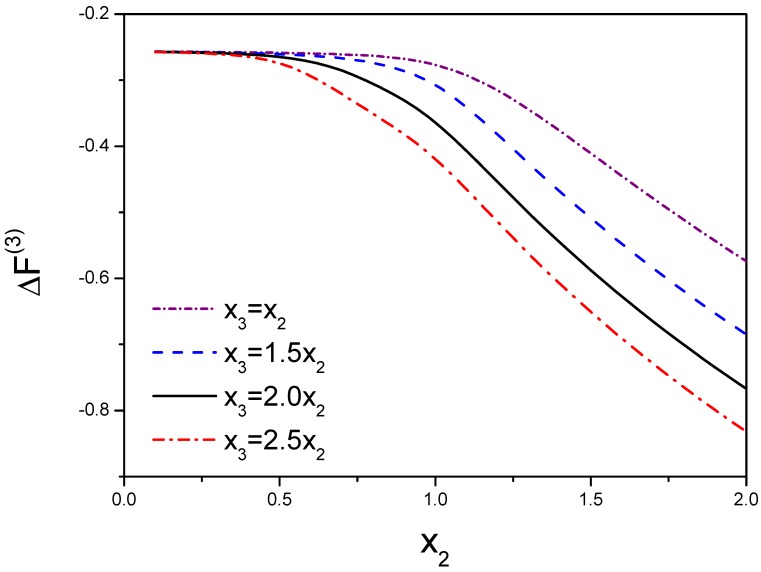
Variation of three molecules’ free energy as a function of the molecular interactions. $x_{2}$ denotes the interactions between molecule 1 and molecule 2. $x_{3}$ is the interaction between the third molecule and dimer. $\Delta F^{\left(3\right)}$ is defined as $F^{\left(3\right)}-3F^{\left(1\right)}$. The competition between molecules results in the variation of the free energy.

In Figure 4, we take $M_{3}=M-3$ and $g_{2}=g_{3}$. This figure demonstrates the free energy and the function of $f^{\left(3\right)}$ are a function of $x_{2}$ and $x_{3}$. Due to $x_{3}=\lambda_{1}x_{2}$, we choose a few of values for $x_{3}=x_{2},1.5x_{2},2x_{2},2.5x_{2}$ that are resulted from the range of $\lambda_{1}$ from 1 to 3.

By using the above equations, we can obtain the landscape of free energy. The parameters such as *g*, σ, *s*, even *z* stand for the different peptide states, and corresponding to coil, helix, β-strand, β-sheet and fibrils. $M_{n}=M-3n$ is used to describe the number of hydrogen bond in the n-th chain. With the change of these parameters, the free energy will be a function as *n*. We have calculated the free energy for n≤6.

**Figure 5:**
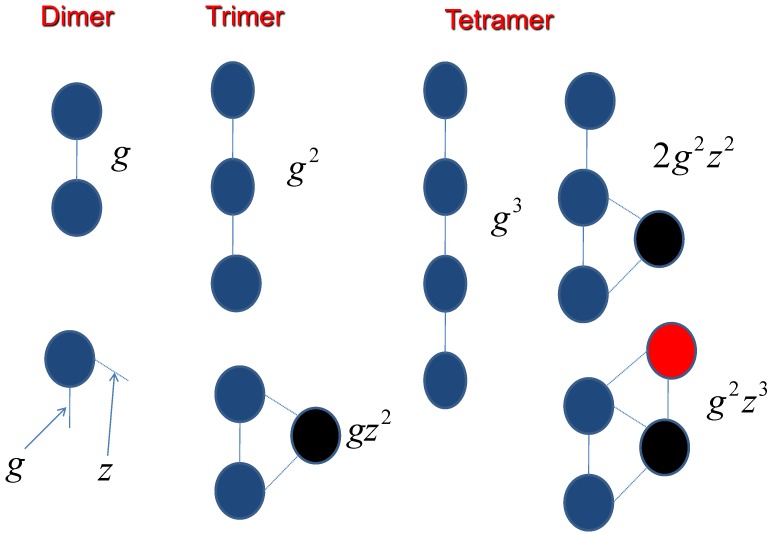
The assembly process is dependent on the molecular interactions.

For counting the side chain effect, the interaction coefficients $g_{n}$ have been demonstrated in Figure 5,

(47) $$\begin{array}{c} g_{2}=g\\ g_{3}=gz\\ g_{4}=g^{2}\left(z+z^{2}\right)\\ g_{5}=g^{3}\left(z+z^{2}+z^{3}\right) \end{array}$$

**Figure 6:**
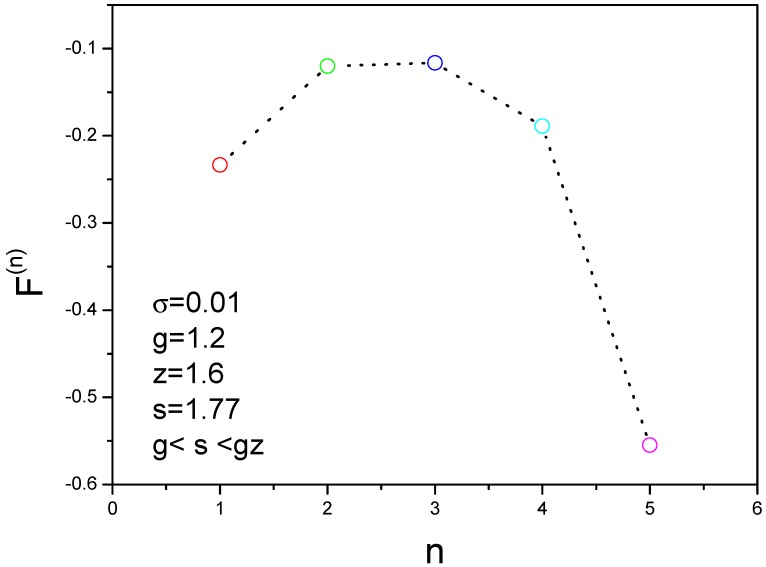
Free energy landscape for different molecules gives a nonmonotonic function that has a peak at three molecules. $F^{\left(n\right)}$ is the free energy of an oligomer containing *n* molecules.

The energy landscape can be obtained as shown in Figure 6, where the parameters are σ=0.01, g=1.2,z=1.6,s=1.77. The nucleation can occur because the condition g<s<gz is satisfied. We find that the free energy has a peak value when *n* is equal to 3. In other words, three molecules have higher free energy and four molecules have lower free energy. This is an important result about the assemble mechanism for aggregation of amyloid fibril. Based on this result, we develop the kinetic formulas to study the nucleation processes of amyloid fibrils.

### 3.3. Peak Location in the 2D Nucleation Rate

A model of three molecules was presented for the dynamics of a nucleating trimer, the nucleation rate in 2D can be written as [31]

(48) $$\begin{array}{c} k_{nuc}=\sum_{x=1}^{L}k_{diff}\varepsilon_{+}\left(x,1\right)C_{2}\left(x\right)\\ =k_{diff}C_{1}^{2}\sum_{x=1}^{L}\varepsilon_{+}\left(x,1\right)\exp\left(-\frac{xf}{kT}\right) \end{array}$$

The analytic solution of $\varepsilon_{+}\left(x,y\right)$ is

(49) $$\varepsilon_{+}\left(x,y\right)=e_{0}\exp\left[-\left(\alpha x+\beta y\right)\right]\tilde{B}\left(x,y\right)$$

where

(50) $$\tilde{B}\left(x,y\right)=\frac{\sum_{m=2}^{\infty }B_{m}\sin\left[m\arctan\left(y/x\right)\right]I_{m}\left(v\sqrt{x^{2}+y^{2}}/2D\right)}{\sum_{m=2}^{\infty }B_{m}\sin\left(m\pi /4\right)I_{m}\left(v\sqrt{2}L/2D\right)}$$

The peak location is determined by the extremum of the nucleation rate,

(51) $$\frac{\partial k_{nuc}}{\partial x}=k_{diff}C_{1}^{2}\sum_{x=1}^{L}\left[\frac{\partial \varepsilon_{+}\left(x,1\right)}{\partial x}-\frac{f}{kT}\varepsilon_{+}\left(x,1\right)\right]\exp\left(-\frac{xf}{kT}\right)=0$$

then

(52) $$\frac{\partial \ln\varepsilon_{+}\left(x,1\right)}{\partial x}=\frac{f}{kT}$$

By using the above equation,

(53) $$\varepsilon_{+}\left(x,1\right)=e_{0}^{\prime }\exp\left(-\alpha x\right)\tilde{B}\left(x,1\right)$$

where $e_{0}^{\prime }=e_{0}\exp\left(-\beta \right)$, $\alpha =\frac{v_{x}}{2D}$, $\beta =\frac{v_{y}}{2D}$.

(54) $$\frac{\partial \tilde{B}\left(x,1\right)}{\partial x}=\left(\varepsilon_{2}+\alpha \right)\tilde{B}\left(x,1\right)$$

where $\varepsilon_{2}=\frac{f}{kT}$.

Because denominator of $\tilde{B}\left(x,1\right)$ is a constant,

(55) $$\frac{\partial {\tilde{B}}_{num}\left(x,1\right)}{\partial x}=\left(\varepsilon_{2}+\alpha \right){\tilde{B}}_{num}\left(x,1\right)$$

where ${\tilde{B}}_{num}$ is a numerator of $\tilde{B}$.

${\tilde{B}}_{num}\left(x,y\right)$ can be rewritten as

(56) $${\tilde{B}}_{num}\left(x,y\right)=\sum_{m=2}^{\infty }B_{m}\sin\left(m\theta \right)I_{m}\left(ar\right)$$

where $r=\sqrt{x^{2}+y^{2}}$, $a=\frac{v_{x}}{2D}$, $v=\sqrt{v_{x}^{2}+v_{y}^{2}}$ and

(57) $$\begin{array}{c} B_{2}=1\\ B_{3}=2\frac{v_{x}-v_{y}}{v}\\ B_{4}=2{\left(\frac{v_{x}-v_{y}}{v}\right)}^{2}\\ B_{5}=2\frac{v_{x}-v_{y}}{v}\\ I_{m}\left(x\right)=\sum_{k=0}^{\infty }\frac{1}{\Gamma (k+1)\Gamma (m+k+1)}{\left(\frac{x}{2}\right)}^{m+2k} \end{array}$$

We take the earlier terms including k=0,1,2,

(58) $$\begin{array}{c} {\tilde{B}}_{num}\left(x,y\right)=\sum_{m=2}B_{m}sin\left(m\theta \right)\sum_{k=0}^{\infty }\frac{{\left(ar\right)}^{2k+m}}{k!\Gamma (k+m+1)}\\ =\sum_{m}B_{m}sin\left(m\theta \right)\left[\frac{{\left(ar\right)}^{m}}{\Gamma (m+1)}+\frac{{\left(ar\right)}^{m+2}}{\Gamma (m+2)}+\frac{{\left(ar\right)}^{m+4}}{2!\Gamma (m+3)}\right] \end{array}$$

By using Γ(n)=(n−1)!, we have

(59) $$\begin{array}{c} {\tilde{B}}_{num}\left(x,y\right)=\frac{a^{2}}{2!}\{r^{2}\sin\left(2\theta \right)+B_{3}\frac{a}{3}r^{3}\sin\left(3\theta \right)+\left[B_{4}\frac{a^{2}}{3\times 4}r^{4}\sin\left(4\theta \right)+\frac{a^{2}}{3}r^{4}\sin\left(2\theta \right)\right]+\\ \left[B_{5}\frac{a^{3}}{3\times 4\times 5}r^{5}\sin\left(5\theta \right)+B_{3}\frac{a^{3}}{3\times 4}r^{5}\sin\left(3\theta \right)]\right\} \end{array}$$

By using $\sin\left(2\theta \right)=2\sin\theta \cos\theta ,\sin\left(3\theta \right)=3\sin\theta -4sin^{3}\theta ,\sin\left(4\theta \right)=4\sin\theta \cos\theta -8\cos\theta \sin^{3}\theta ,\sin\left(5\theta \right)=5\sin\theta -20\sin^{3}\theta +16\sin^{5}\theta$, x=rsinθ, y=rcosθ, we get

(60) $$\begin{array}{c} {\tilde{B}}_{num}\left(x,y\right)=\frac{a^{2}}{2!}[\left(B_{5}\frac{a^{3}}{12}+B_{3}\frac{a^{3}}{4}\right)x^{4}y+\left(B_{4}\frac{a^{2}}{3}+\frac{2a^{2}}{3}\right)x^{3}y\\ +aB_{3}x^{2}y+\left(-B_{5}\frac{a^{3}}{6}+B_{3}\frac{a^{3}}{6}\right)x^{2}y^{3}+2xy+\left(-B_{4}\frac{a^{2}}{3}+\frac{2a^{2}}{3}\right)xy^{3}\\ -B_{3}\frac{a}{3}y^{3}+\left(B_{5}\frac{a^{3}}{60}-B_{3}\frac{a^{3}}{12}\right)y^{5}] \end{array}$$

The coefficients can be defined as

(61) $$\begin{array}{c} f_{41}=\left(B_{5}+3B_{3}\right)\frac{a^{3}}{12}=\left(\alpha -\beta \right)\frac{a^{2}}{3}\\ f_{31}=\left(B_{4}+2\right)\frac{a^{2}}{3}=\frac{1}{3}\left(4a^{2}-\alpha \beta \right)\\ f_{21}=aB_{3}=\alpha -\beta \\ f_{23}=\left(-B_{5}+B_{3}\right)\frac{a^{3}}{6}\\ f_{11}=2\\ f_{13}=\left(-B_{4}+2\right)\frac{a^{2}}{3}=\frac{\alpha \beta }{3}\\ f_{03}=-B_{3}\frac{a}{3}=-\frac{\alpha -\beta }{3}\\ f_{05}=\left(B_{5}-5B_{3}\right)\frac{a^{3}}{60}=-\frac{a^{2}}{15}\left(\alpha -\beta \right) \end{array}$$

thus

(62) $$\begin{array}{c} \tilde{B}\left(x,y\right)=f_{41}x^{4}y+f_{31}x^{3}y+f_{21}x^{2}y\\ +f_{23}x^{2}y^{3}+f_{11}xy+f_{13}xy^{3}+f_{03}y^{3}+f_{05}y^{5} \end{array}$$

When y=1, the above expression can be written as

(63) $${\tilde{B}}_{num}\left(x,1\right)=b_{4}x^{4}+b_{3}x^{3}+b_{2}x^{2}+b_{1}x+b_{0}$$

where

(64) $$\begin{array}{c} b_{4}=f_{41}=\left(\alpha -\beta \right)\frac{a^{2}}{3}\\ b_{3}=f_{31}=\frac{1}{3}\left(4a^{2}-\alpha \beta \right)\\ b_{2}=f_{21}+f_{23}=\alpha -\beta \\ b_{1}=f_{11}+f_{13}=2+\frac{\alpha \beta }{3}\\ b_{0}=f_{03}+f_{05}=-\frac{\alpha -\beta }{3}-\frac{a^{2}}{15}\left(\alpha -\beta \right)=-\frac{1}{15}\left(\alpha -\beta \right)\left(a^{2}+5\right) \end{array}$$

The expressions of $v_{x}$ and $v_{y}$ are employed by

(65) $$\begin{array}{c} v_{x}=2\frac{\left[\exp(-Eweak)-1\right]}{\exp(-Eweak)+1}\\ v_{y}=2\frac{\left[\exp(-Estrong)-1\right]}{\exp(-Estrong)+1} \end{array}$$

where $Eweak=\varepsilon_{2}$.

We will discuss three cases for taking different $\varepsilon_{2}$ to calculate the coefficients of polynomial,

**Case I:**$\varepsilon_{2}=0.2$, Estrong=−0.5

(66) $$\begin{array}{c} b_{4}=-0.00200776\\ b_{3}=0.0314431\\ b_{2}=-0.344587\\ b_{1}=1.99186\\ b_{0}=0.115264 \end{array}$$

then inserting these coefficients and obtain the peak value of *x*,

(67) $$x_{m}^{\varepsilon_{2}=0.2}=3.5$$

which is in agreement with the numerical result $x_{m}^{\varepsilon_{2}=0.2}=4$ [31].

**Case II:**$\varepsilon_{2}=0.3$, Estrong=−0.5

(68) $$\begin{array}{c} b_{4}=-0.00269598\\ b_{3}=0.0395389\\ b_{2}=-0.393804\\ b_{1}=1.98785\\ b_{0}=0.131807 \end{array}$$

then inserting these coefficients and obtain the peak value of *x*,

(69) $$x_{m}^{\varepsilon_{2}=0.3}=2.8$$

which is in agreement with the numerical result $x_{m}^{\varepsilon_{2}=0.2}=3$ [31].

**Case III:**$\varepsilon_{2}=0.4$, Estrong=−0.5

(70) $$\begin{array}{c} b_{4}=-0.00364679\\ b_{3}=0.0490944\\ b_{2}=-0.442294\\ b_{1}=1.98389\\ b_{0}=0.148161 \end{array}$$

then inserting these coefficients and obtain the peak value of *x*,

(71) $$x_{m}^{\varepsilon_{2}=0.3}=2.2$$

which is in agreement with the numerical result $x_{m}^{\varepsilon_{2}=0.2}=2$ [31].

It is reasonable to neglect the higher terms from the above data, i.e., $b_{4}=0$ and $b_{3}=0$, so the polynomial is changed to

(72) $${\tilde{B}}_{num}\left(x,1\right)=b_{2}x^{2}+b_{1}x+b_{0}$$

Due to $b_{1}^{2}>>4b_{0}b_{2},b_{2}^{2}$, and consider *x* is a positive number, so the root of equation is simplified as

(73) $$x_{m}\approx \frac{1}{\varepsilon_{2}+\alpha }$$

The nucleation flux is a nonmonotonic function of the number of the hydrogen bonds in dimer when the third molecule is added to form trimer. The peak values are a significant result of the deviation of the lowest free energy pathway.

### 3.4. Scaling Behavior of Nucleation Rate in 3D

The system of three molecules is unstable, so we have developed a stable 3D model. The nucleation rate in 3D model can be written as [32]

(74) $$k_{nuc}=\sum_{x=1}^{L}k_{diff}\varepsilon_{mol}\left(x\right)C_{2}\left(x\right)$$

where

(75) $$\varepsilon_{mol}\left(x\right)=\frac{{\left(\frac{1}{p_{+mol}}-1\right)}^{x}-1}{{\left(\frac{1}{p_{+mol}}-1\right)}^{n^{⋆}}-1}$$

and

(76) $$C_{2}\left(x\right)=C_{1}^{2}exp\left(-xf/kT\right)$$

$p_{+mol}$ can be defined as

(77) $$p_{+mol}=\frac{k_{diff}}{k_{diff}+k_{loss}}$$

Inserting the above equation into $k_{nuc}$, we have

(78) $$k_{nuc}=\sum_{x=1}^{L}k_{diff}\frac{{\left(\frac{k_{loss}}{k_{diff}}\right)}^{x}-1}{{\left(\frac{k_{loss}}{k_{diff}}\right)}^{n^{⋆}}-1}C_{1}^{2}\exp\left(-xf/kT\right)$$

furthermore,

(79) $$k_{nuc}=\frac{k_{diff}C_{1}^{2}}{{\left(\frac{k_{loss}}{k_{diff}}\right)}^{n^{⋆}}-1}\sum_{x=1}^{L}\left\{{\left[\frac{k_{loss}}{k_{diff}}\exp\left(-\frac{f}{kT}\right)\right]}^{x}-{\left[\exp\left(-\frac{f}{kT}\right)\right]}^{x}\right\}$$

With the help of

(80) $$\sum_{x=1}^{L}y^{x}=\frac{y^{L+1}-y}{y-1}$$

then

(81) $$\begin{array}{c} k_{nuc}=\frac{k_{diff}C_{1}^{2}}{{\left(\frac{k_{loss}}{k_{diff}}\right)}^{n^{⋆}}-1}\exp\left(-\frac{f}{kT}\right)\{\frac{k_{loss}}{k_{diff}}\frac{{\left(\frac{k_{loss}}{k_{diff}}\right)}^{L}\exp\left(-\frac{fL}{kT}\right)-1}{\frac{k_{loss}}{k_{diff}}\exp\left(-\frac{f}{kT}\right)-1}\\ -\frac{\exp{\left(-\frac{f}{kT}\right)}^{L}-1}{\exp(-\frac{f}{kT})-1}\} \end{array}$$

Based on the numerical result [32], we will discuss f>0.

When f>0, $1>\exp\left(-\frac{f}{kT}\right)>>\exp\left(-\frac{fL}{kT}\right)$. In this case, the first approximation is $\exp(-\frac{fL}{kT})\to 0$

(82) $$k_{nuc}=\frac{k_{diff}C_{1}^{2}}{{\left(\frac{k_{loss}}{k_{diff}}\right)}^{n^{⋆}}-1}\left\{\frac{1}{1-\exp(\frac{f}{kT})}-\frac{k_{loss}/k_{diff}}{k_{loss}/k_{diff}-\exp\left(\frac{f}{kT}\right)}\right\}$$

In the condition of $\frac{k_{loss}}{k_{diff}}>1$

(83) $$k_{nuc}\approx \frac{k_{diff}^{n^{⋆}}C_{1}^{2}}{k_{loss}^{n^{⋆}-1}}\exp\left(-\frac{f}{kT}\right)$$

Due to $k_{diff}\propto C_{1}$, so the scaling behavior in this case is

(84) $$k_{nuc}\propto C_{1}^{n^{⋆}+2}$$

This condition matches the numerical result, so the analytic result can be written as

(85) $$Logk_{nuc}=\left(n^{⋆}+2\right)LogC_{0}+const$$

where $C_{1}=C_{0}$ and const is a constant including *f* and other terms.

In the experimental measurement of the nucleation process [33,34,35], kinetics were monitored by ThT fluorescence. The solution conditions for fibril formation were 100 mM KCl, 50 mM potassium phosphate, PH 7.4, 25 °C. Fiber sample were prepared as for transmission electron microscopy. The experimental result is $k_{nuc}\propto 4$. When we take $n^{⋆}=2$ in the analytic result, the theoretical result is in agreement with the experimental result.

## 4. Conclusions

In summary, a microscopic model with three states including coil, helix and sheet is constructed to explore the mechanism of amyloid formation. The partition function and thermodynamic quantities of many molecule systems are obtained by considering the repulsive and attractive interactions. The equilibrium properties of the system in the solution have been investigated. Free energy landscape and side chain effect are illustrated. It is found that the system of three molecules has higher free energy. The kinetic properties of molecules related with amyloid formation are also studied. By using the random walk model in 2D and 3D, the nucleation processes of amyloid fibrils are quantitatively demonstrated. The microscopic theoretical model and results can be in agreement with numerical and experimental results. These theoretical approaches of the microscopic model could be used to improve the computational simulations in new timescale.
